# Ecological and environmental conditions correlate with flea population within human habitations in a plague focus, Mbulu district, Tanzania

**DOI:** 10.1371/journal.pgph.0004751

**Published:** 2025-06-18

**Authors:** Stella T. Kessy, Alfan A. Rija

**Affiliations:** 1 School of Life Science and Bio-Engineering (LiSBE), Nelson Mandela African Institution of Science and Technology, Arusha, Tanzania; 2 Department of Wildlife Management, Sokoine University of Agriculture, CHUO KIKUU, Morogoro, Tanzania; Tunisian Institute of Veterinary Research, TUNISIA

## Abstract

Plague persistence remains a major health concern in some African countries. Although the role of some flea vectors in their transmission is widely known, the local-scale factors contributing to human plague recurrence are still poorly understood, thwarting the potential disease mitigation measures in affected communities. We examined the flea population and their relationship to household practices in rural Mbulu District, northern Tanzania. We sampled fleas from both host (170 rodents in 3 species) and household interiors using a Light trap (Ltrap). We found seven species of flea inhabiting the human dwelling, with *Xenopsylla brasilliensis* the most common species occupying rodent species and the in-house environment. *Dinopsylla lypusus* and three others: *Ctenocephalides canis*, *Ctenocephalides felis* and *Xenopsylla cheopis*, partitioned between the rodents and the house environment, respectively, indicating niche separation and distinct disease transmission risks in these vectors. Further, the flea index varied throughout the sampling period, with the total flea index exceeding the threshold of 1. We observed that *X. brasilliensis* (mean = 1.93 ± 0.56SE, p < 0.001) significantly increased the abundance of rodent fleas. Furthermore, households without keeping livestock under the same roof (mean = - 0.97 ± 0.16SE, p < 0.001) and not owning livestock (mean = -1. 45 ± 0.31SE, p < 0.001) had a higher likelihood of decreased house flea population than households sharing livestock under one roof. Similarly, houses with bed arrangements with a sleeping loft positively correlated with increased rodent flea abundance (mean = 1.88 ± 1.04SE, p = 0.07), strongly suggesting the importance of improving rural lifestyle. Enhancing public education on hygiene and flea control measures to reduce the risks of plague persistence and transmission in these rural communities is an increasing priority.

## Introduction

Fleas are parasitic insects that are known to infest a variety of mammals, including humans, dogs, and cats [1,2]. They are highly mobile, able to jump up to 150 times their own body length, allowing them to move quickly between hosts. They can also survive for long periods without a host, and their pupae can remain viable until suitable conditions for development are met, allowing them to persist in the environment [3]. Furthermore, fleas serve as vectors of many pathogens (e.g., *Yersinia pestis* [4], *Rickettsia typhi* [5] and *Bartonella henselae)* that can be transmitted between animals and humans, establishing a cycle of disease transmission in both populations. Understanding the biology and behaviour of fleas and how environmental conditions and human lifestyles shape their population dynamics and disease transmission risks is crucial for improving the strategies for reducing infection risks to both animals and humans. Unfortunately, such data is missing for most rural communities facing plague (a disease transmitted by fleas) risks, endangering the public health.

Flea abundance is among the factors that influence plague disease transmission, and is influenced by several factors including host availability, temperature, humidity, and habitat characteristics [6–9]. When the temperature is very low, for example, flea development slows down, but flea growth and development peaks within specific temperature thresholds, which vary between species, resulting in rapid population growth under optimal conditions [10]. However, flea population may respond to species extrinsic factors such as host density. For example, during the rainy season with warm temperatures, vegetation production increases, thereby increasing rodent densities, which favours flea populations due to available food and shelter [11]. Moreover, previous studies have documented host preferences of different flea species [12,13] including a variety of rodent hosts which are the primary hosts of plague [14] suggesting high dynamics and risks of disease transmission. The human flea (*Pulex irritans*), for example, is known to prefer humans as hosts, but can also infest a range of other animals, including dogs, cats, pigs, and rodents [15] making it an agent of zoonoses. Similarly, the cat flea (*Ctenocephalides felis*) and the dog flea (*Ctenocephalides canis*), are more commonly found on their respective specific hosts, but also can infest other animals and even humans [16–19]. In this case, the complex interplay of host preference, environmental conditions, and habitat characteristics greatly impacts the dynamics and distribution of flea populations.

Previous studies that have looked into the relationship between household behavior and flea abundance have found that households with poor sanitation and hygiene practices are more likely to have higher flea abundance due to increased food and shelter availability for fleas [20,21]. This is because, flea immature stages feed on organic debris on carpets, furniture or soil [22,23], and use cluttered or poorly maintained areas as favorable habitats, such as poorly maintained walls or floor and domestic animal resting places [24–26]. Also, fleas may be attracted to the warmth and moisture generated by certain household furniture, such as carpets, curtains, beddings as well as certain house designs such as thatched roof mud wall houses [27–29]. Overall, these attributes promote flea survival and infestation while also increasing the possibility of fleas, human and other host interactions and increasing the propensity for flea borne diseases such as plague to persist in the foci.

In Tanzania, plague is a potentially re-emerging disease since it has been intermittent since precolonial time [30–33]. The largest plague outbreak occurred in Lushoto district in 1980 claiming 640 human lives over 13 years of its persistence [34,35]. This was followed by other outbreaks in Karatu and Mbulu District in 1996 [34,36]. Further, studies indicate that all of these Tanzanian foci that have undergone recurring disease outbreaks, have resulted into a substantial number of human cases and case-fatality rates. Mbulu district for instance, has recently reported a substantial number of human plague cases [37,38]. Also, plague pathogen is known to occur in rodent reservoirs and vectors which contributes to the transmission and persistence of plague in the foci [37–41]. Flea species, such as *Xenopsylla cheopis* and *Xenopsylla brasilliensis*, were observed in domestic environments on *Ratus rattus*, in semi domestic on *Mastomys natalensis* and in wild environments on *Lemniscomys zebra* [40]. Land use change has also been found to influence dynamics of plague transmission in the foci [42]. Studies in Lushoto district, showed the sporadic outbreaks of plague were linked to poor housing and environmental sanitation [34,36] but this may vary at local and spatial scales due to varying conditions that predict disease re-occurrence. [40]

In this study, we aimed to examine the dynamics of flea index across the sampling period and determine how household sleeping behavior (such as mat and sleeping loft) influence flea abundance within houses in a plague focus of Mbulu District in northern Tanzania. Specifically, we aimed to understand (i) the relationships between rodent host abundance and flea species distribution across different flea sources inside houses, (ii) the variation in flea index between various sources across the sampling period, (iii) how environmental factors (season, rainfall, temperature and humidity) and flea species identity influence flea abundance and (iv) effect of household behavior or practices on flea abundance. We hypothesized that flea index within houses would vary during the course of the sampling period due to changes in seasonality and presence of flea refugia such as rodents. Additionally, we hypothesized that, flea abundance would be higher in the dry season than other seasons due to higher temperature which favor flea growth and development and some flea species such as *P. irritans*, *C. canis* and *C. felis* would be more abundant due to diverse host choices within houses. Furthermore, we predicted that houses with poor sanitation and hygiene practices such as keeping livestock within the sleeping house, will show higher probability of having more fleas due to increased availability of surplus food and shelter for fleas and other hosts such as rodents. This information may be useful in designing the appropriate strategies to reduce flea predisposing causes, thereby reducing the likelihood of a plague outbreak in these rural communities.

## Materials and methods

### Ethics statement

The study was approved by the institutional Review Board of Sokoine University of Agriculture, Tanzania (Ref. No. SUA/DPRTC/R/186 VOL.II), Manyara region Ref. no FA.262/347/01/H/247 and Mbulu District Ref. no AB.323/381/01/`B`/9. Before beginning experiments in the selected houses, we explained the purpose of the research to the participant and confirmed written consent from each participant. Animal handling followed the guidelines of the American Society of Mammalogists (ASM) for the use of mammals in research and education (Sikes and Animal Care and Use Committee of the American Society of Mammalogists, 2016)

### Study area

**Fig 1:**
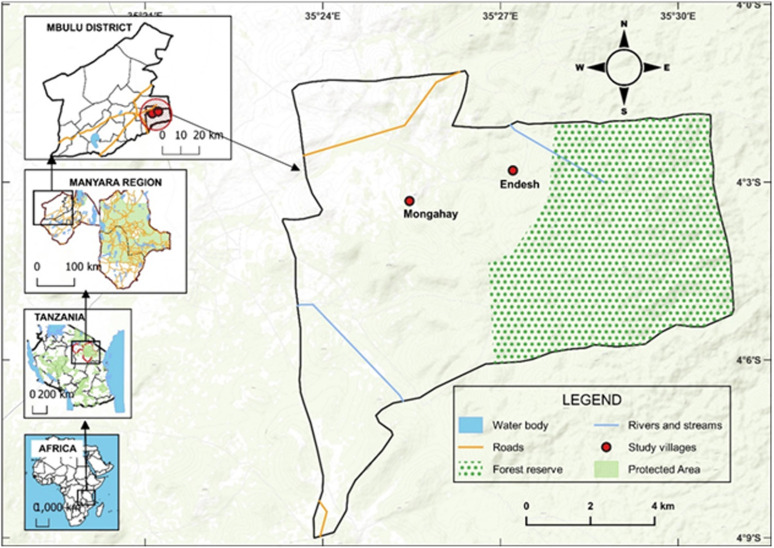
Map of Mbulu District in Tanzania showing the locations of the study sites Endeshi and Mongahay villages. Map was generated using QGIS software (version 3.8.3 Zanzibar). Base layers were sourced from Esri map; https://esri.maps.arcgis.com/home/item.html?id=273ffd9a4c054d47843ed9642ecb143e, licensed under the Esri Master license agreement; https://goto.arcgis.com/termsofuse/viewtermsofuse.

The study was conducted between January to December 2019 in Mbulu district, in Northern Tanzania, at 3^°^ 57′ 097″S, 35^°^ 18′ 39.60″E (Fig 1). The district extends between1000–2400 m above sea level, and has a semi-arid to sub-humid condition with biannual rainfall ranging from 400mm to 1200mm between March and May, and between October to December respectively. The economic activities are primarily livestock keeping and agriculture. Two villages Endesh and Mongahay, were randomly chosen for the study due to the history of plague outbreaks in the last 12 years following previous detection of plague bacteria in rodents and sporadic human plague cases suggesting that the bacteria are persisting and circulating in these areas [34,36,40,43].

### House selection and the assessment of household environments and practices

**Fig 2:**
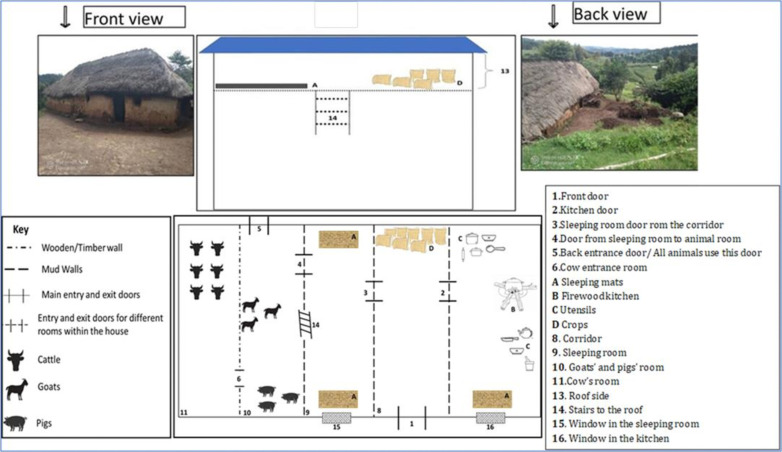
Illustration of house designs outside and inside houses in the study community. Most houses had thatched and corrugated roof with mud walls and mud floor. Some had a loft chamber for sleeping and/ or storing crops with livestock often kept in corals near the house and/ or inside same sleeping houses. [47].

A randomly sampled group of 44 houses were selected for rodent trapping, flea collection and household assessment. Before assessment, we obtained verbal consent from the owner of the house through a clear and transparent explanation of the observation process. A mutual agreement was reached regarding the recording of household characteristics for the purpose of understanding how human practices and house surroundings could influence flea and rodent abundance in this rural setting [36,44–46]. The factors measured included the; (i) presence/ absence of livestock, presence of livestock in the vicinity and their specific locations, specifically identifying coral areas, whether located inside or outside houses, (ii) crop storage behavior by recoding areas used for stored and their proximity to living areas, (iii) House styles including house type (brick with thatched roof, brick with corrugated iron sheet roof, mud with thatched roof, mud with corrugated iron sheet roof), (iv) floor type (mud, cement), (v) wall type (mud, plastered with bricks),and (vi) sleeping arrangement observing the bedding available in the household (bed type mattress, mats or loft room where a person sleeps with a mat) (Fig 2).

### Flea collection

To collect fleas from rodents, animals were live trapped using Sherman traps and locally made wooden box traps (11 cm width, 17 cm length and 16 cm height size). The locally wooded box trap is rectangular made up of wire mesh and small pieces of wood. It is operated by luring the rodent inside to reach the bait. Once the rodent is triggered, the trap door closes behind without harming the animal. These traps have been used in different studies to capture rodents inside houses especially the species such as *Ratus rattus* [48]. Both traps were baited with peanut butter mixed with maize flour and left overnight and inspected each morning for three consecutive days. Captured animals were anaesthetized with diethyl ether for immobilization [49]. All these procedures were conducted in accordance with institutional animal care guidelines to minimize stress and discomfort to the animal [50]. Other morphological characteristics were recorded such as weight, sex and reproductive status and rodents were identified to species level using relevant keys [51,52] and confirmed by sequencing the mitochondrial cytochrome b gene at Czech Republic, Institute of Vertebrate Biology, and have been reported elsewhere [53].

To collect fleas from the indoor environment, one light trap (Ltrap) was installed in each house where rodents were also captured. The Ltrap consisted of a flash light and a metal tray quarter filled with water. Vaseline jelly was smeared to inner edge of the tray to prevent fleas from escaping. The flash light was directed to the center of the tray to successfully attract fleas, taking advantage of flea’s known phototropic behavior. This design was chosen to emulate the conditions that have been shown to maximize capture rates [54–56]. However, in the morning when the traps were removed, the flashlight was switched off. Fleas captured overnight were removed from the tray in the morning.

Further, fleas were sorted according to villages, month, and whether they were collected from the rodents or house floor, counted and preserved in 70% ethanol for identification in the laboratory. The fleas were identified to genus and/or species level, following the methods described in [57,58]. The fleas were then processed by adopting a modified version of the method described in [59]. Briefly, each pool from the sorted groups was exposed to NaOH 10%, dehydrated in various concentrations of ethanol (50%, 70%, 95%, absolute), cleared in clove oil, temporarily mounted using glycerin on a microscopic slide, and examined under a light microscope using a 10x objective. The processes were applied to improve flea visibility, preserve flea morphology for proper analysis and enable examination under a light microscope. Both rodent captures and flea collection were conducted from January 2019 to December 2019.

#### Data analysis.

To determine the relative abundance of the rodent species, we used trap success percentage using the following formula:


$$\mathrm{\backslash [}Trapsucess=\;\frac{N}{N_{t}xNn}\times 100\mathrm{\backslash ]}$$

where, N = total number of rodents trapped, Nt = number of traps used per night, Nn = total number of trapping nights.

To summarize the information on the distribution of the flea species from the flea source we used descriptive statistics and obtained the total flea index from the rodent flea using the formula:

Total flea index = (total number of fleas from rodents/ total number of rodents captured). Rodent species X flea index = (total number of fleas from rodent X/ total number of rodent X captured). For the house fleas (Ltrap); Total flea index = (total number of fleas collected from the trap/ number of traps). Percentage of flea species Y = (Number of flea species Y from a flea source/ Total number of fleas collected from that flea source) × 100.

Percentage of rodent fleas collected from different household factors = (Number of fleas collected from specific house type X/ total number of rodent fleas in all house types) × 100.

To assess how the flea index from the rodent hosts and the Ltrap fluctuated throughout the sampling period and to understand pattern of flea distribution within each season, flea index for each month was analyzed using the above formulas. We plotted flea indices to visualize their dynamics across the sampling period.

Further, flea abundance was referred as the total number of fleas collected from the rodents and Ltrap during the sampling period regardless of the species identity as they constituted the inside flea pool potential threat to humans. To explore the relationship between rodent abundance and the house flea abundance, we used Spearman’s rank correlation. This was selected after the confirmation using Shapiro-Wilk test that both variables were not normally distributed (p < 0.05). Then ggscatter function from the ggpubr package was used to create a scatter plot with a regression line to highlight this relationship trend and a 95% confidence interval for better clarity.

To assess how environmental factors and flea species identity, influence the rodent flea abundance as well as the house flea (Ltrap flea), we built a generalized linear model (GLM) with negative binomial error distribution. This was used after confirming data overdispersion using DHARMa package. Prior to modelling, we examined multicollinearity between temperature, humidity and rainfall variables. We chose to include temperature and rainfall in the models and dropped humidity due to high correlation between Temperature and humidity (r = -0.8). Temperature is known to influence flea growth and development [10,60]. The first model included temperature, rainfall, flea species and season as fixed factors. The relative influence of each variable in the model was evaluated by deleting the non-significant model term in a backward step-wise process, assessing model variance at each step of the modelling. The best model fitting the data was chosen using the Akaike Information Criteria (AIC). To assess the model’s goodness of fit, we generated a four-panel plot that included a residuals histogram, a Q-Q plot of standardized deviance residuals, a plot of fitted values versus residuals, and a plot of Cook’s distance. These diagnostic plots allowed us to assess the assumptions of the model, identify influential observations, and ensure the model’s validity.

Further, to examine the effect of house characteristics and human behavior on flea abundance, we build generalized liner model (GLM) with a negative binomial error distribution for both rodent fleas and the house flea data, after confirming overdispersion. We first built a global model that includes six variables: house type, floor type, wall type, crop storage, cattle keeping and sleeping arrangement as predictors. The Wald test was used to examine model significance [61]Using a step-wise model deletion process as above.

Finally, a predictive model was used to assess the relative effect of each significant model term in the final plausible model. We visualised each model’s model performance using *ggplot2*. All data analyses were conducted in the R program, version 4.3.1. The data and R codes underlying the analysis are found in link [62].

## Results

### Host abundance and distribution of flea species

A total of 170 rodent host belonging to 3 species were captured in 1584 trap nights with 10.73% trap success. Of the captured species the highest abundant was *Ratus rattus* (N = 133; trap success = 8.40%), followed by *Mastomys natalensis* (N = 33; trap success = 2.08%) and *Aethomys kaiseri* (N = 4; trap success = 0.25%). Further, 1034 fleas recorded from seven species exhibited varied distribution across different sources (Table 1).

**Table 1:** Distribution of flea species N (%) across the hosts.

Flea source	*C. canis*	*C. felis*	*D. lypusus*	*E. gallinacea*	*P. irritans*	*X. brasiliensis*	*X. cheopis*
*A. kaiseri*	0	0	3 (50)	0	0	3 (50)	0
*M. natalensis*	0	0	10 (27.7)	0	0	26 (72.2)	0
*R. rattus*	12 (7.36)	10 (6.13)	25 (15.33)	0	62 (38.03)	54 (33.13)	0
Ltrap	263 (31.72)	166 (20.02)	0	147 (17.73)	207 (24.97)	43 (5.19)	3 (36)

Majority of the flea species (N = 829; 80.17%) were collected from the Ltrap and least from the rodent host (N = 205; 19.83%). Fleas appeared to vary in number across the rodent species from *R. rattus (*N = 163; 79.51%), *M. natalensis* (N = 36; 17.56%) and *A. kaiseri* (N = 6; 2.93%). The dominant flea species *Xenopsylla brasiliensis* was collected on both rodent species and the Ltrap. Three flea species *C. canis*, *C. felis* and *X. cheopis* were caught in the Ltrap while one species *Dinopsyllus lypusus s* was collected only on the rodent species. Furthermore, we found highest flea abundance in the houses with mud wall type, with 196 fleas (95.6%) collected from rodents and 795 fleas from Ltrap (96%). Similarly, in house with mud floor type, we observed 198 from rodents (97%) and 810 fleas from Ltrap (98%) (Table 2).

**Table 2:** Distribution of rodent flea and house flea N (%) by household attributes.

Household factors	Number of Household	Rodent Flea	House flea (Ltrap)
**House type**			
brick and thatched roof	1	3(1)	12(1)
brick with corrugated roof	4	7(3)	25(3)
mud thatched roof	20	98(47)	497(60)
mud corrugated roof	19	97(47)	295(36)
**Wall type**			
Mud	39	196(95.6)	795(96)
plastered with bricks	5	9(4)	34(4)
**Floor type**			
Mud	41	198(97)	810(98)
Cement	3	7(3)	19(2)
**Sleeping arrangement**			
Bed and mat	14	51(25)	240(29)
Bed and mat/mattress	18	76(37)	386(47)
Bed and mattress	10	27(13)	101(12)
Bed and mat/mattress as well as a loft	2	23	102(12)
**Livestock keeping**			
Inside household	19	133(65)	565(68)
Outside household	21	62(30)	236(28)
No livestock	4	10(5)	28(3)
**Crop storage**			
Inside household	33	170(83)	636(77)
Outside household	11	35(12)	193(23)

### Flea index across the sampling period

**Fig 3:**
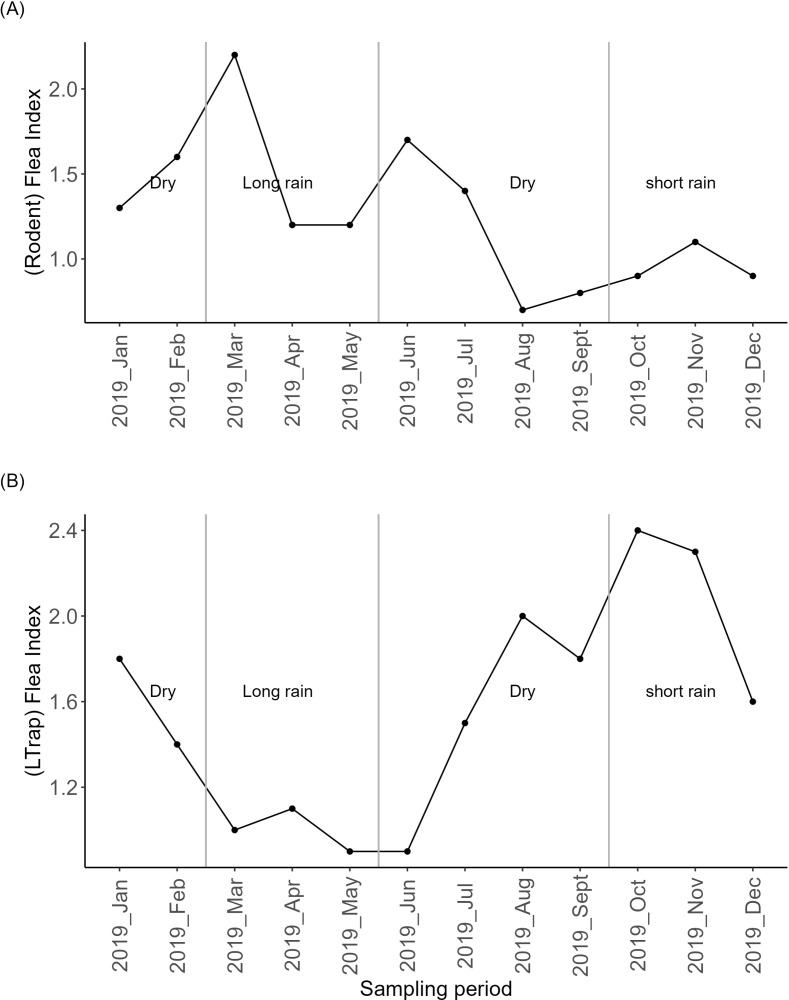
Rodent flea index (A) and Ltrap flea index (B) across the sampling period.

The total flea index of the rodent fleas was 1.2, with the highest index found on rodent species *A. kaiseri* 1.5, followed by *R.rattus* 1.2 and *M. natalensis* 1.1. The flea index varied substantially during the sampling period, increased at the beginning of each season. Specifically, higher flea index was observed in the months of March, June and November (Fig 3A). Further, the Ltrap flea had a total flea index of 1.6 and varied during the sampling period, exhibited low index during the long rain season and increased from June during the dry season and reached its peak in October, before decreasing in November during the short rain season (Fig 3B).

Furthermore, flea index showed varying patterns from the sources and seasons (Table 3). During the dry season, the rodent species *M. natalensis* had the highest total flea index of 1.8 than other rodent species and was mostly contributed by *Xenopsylla brasilliensis*.In contrast, the Ltrap had a total flea index of 9, with flea species *Ctenocephalides canis* and *Pulex irritans* contributing the highest indices of 3.2 and 3.0 respectively.

**Table 3:** Flea index from the various sources and pattern of flea species occurrence across the sampled seasons.

Seasons	Flea source	*C. canis*	*C. felis*	*D. lypusus*	*E. gallinacea*	*P. irritans*	*X. brasiliensis*	*X. cheopis*	Total flea index
Dry	*M.natalensis*	0	0	0.6	0	0	1.2	0	1.8
	*R.rattus*	0.1	0.1	0.2	0	0.4	0.3	0	1
	*A.kaiseri*	0	0	0	0	0	0	0	0
	Ltrap	3.2	0.8	0	1.8	3	0.5	0	9
Long rain	*M.natalensis*	0	0	0.5	0	0	0.8	0	0.4
	*R.rattus*	0.1	0.03	0.2	0	0.8	0.6	0	1.7
	*A.kaiseri*	0	0	0	0	0	0	0	0
	Ltrap	0.6	1.3	0	0.1	0.8	0.3	0	3
Short rain	*M.natalensis*	0	0	0.1	0	0	0.6	0	0.6
	*R.rattus*	0	0	0.2	0	0.2	0.3	0	0.7
	*A.kaiseri*	0	0	0.8	0	0	0.8	0	1.5
	Ltrap	2.2	1.6	0	1.4	0.9	0.2	0.1	6.4
* Flea index 0 means the flea species was not captured in that season									

The pattern, however, changed during the long rain season, where the rodent species *R.rattus* showed the highest total flea index of 1.7 over other rodent species. Similarly, during the same season *P.irritans* and *X.brasilliensis* recorded the highest flea indices of 0.8 and 0.6 respectively. On the Ltrap, we found three times lower flea index than the dry season, with the flea species *Ctenocephalides felis* having relatively lower flea index of 1.3 than for the dry season. Also, during the short rain season, the rodent species *A.kaiseri* had the highest total flea index of 1.5 than other rodent species which was associated with the flea species *Dinopsyllus lypusus* and *X.brasilliensis* both of which had a flea index of 0.8 recorded from the same rodent species. In the Ltrap the total flea index was found to be 6.4, with flea species *C. canis*, *C. felis* and *Echidnophaga gallinacean* having the highest flea index of 2.2, 1.6 and 1.4 respectively.

### Relationship between rodent and House flea abundance

**Fig 4:**
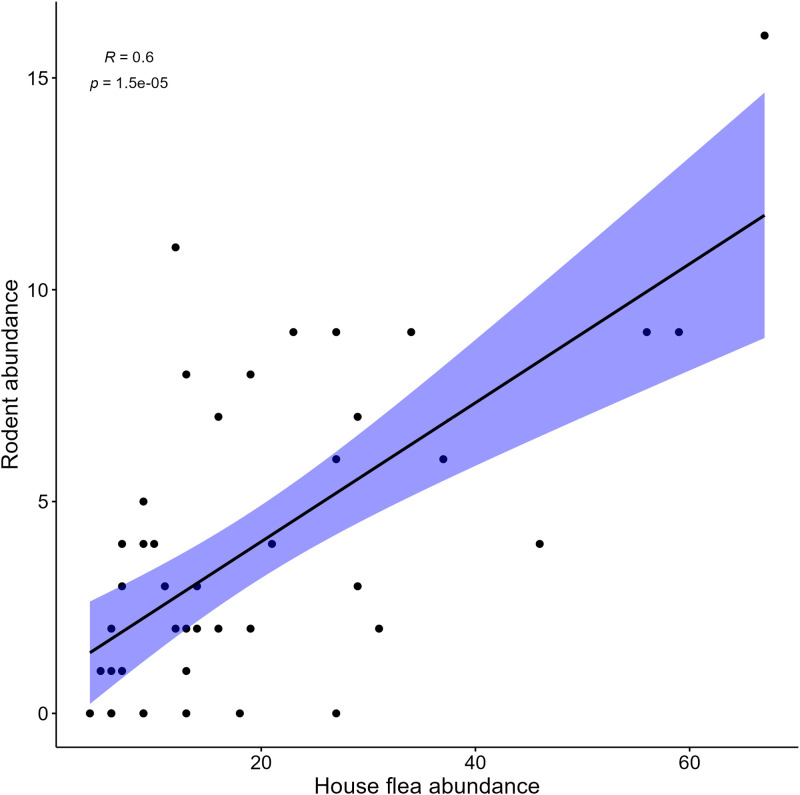
Scatter plot showing the relationship between rodent abundance and the house flea abundance.

There was a significant positive relationship between rodent and house flea abundance, with a correlation coefficient *R* = 0.6; p < 0.001 (Fig 4). This indicates that as rodent abundance increases, house flea abundance also tends to increase.

#### Influence of season and flea species on rodent flea and house flea abundance.

**Fig 5:**
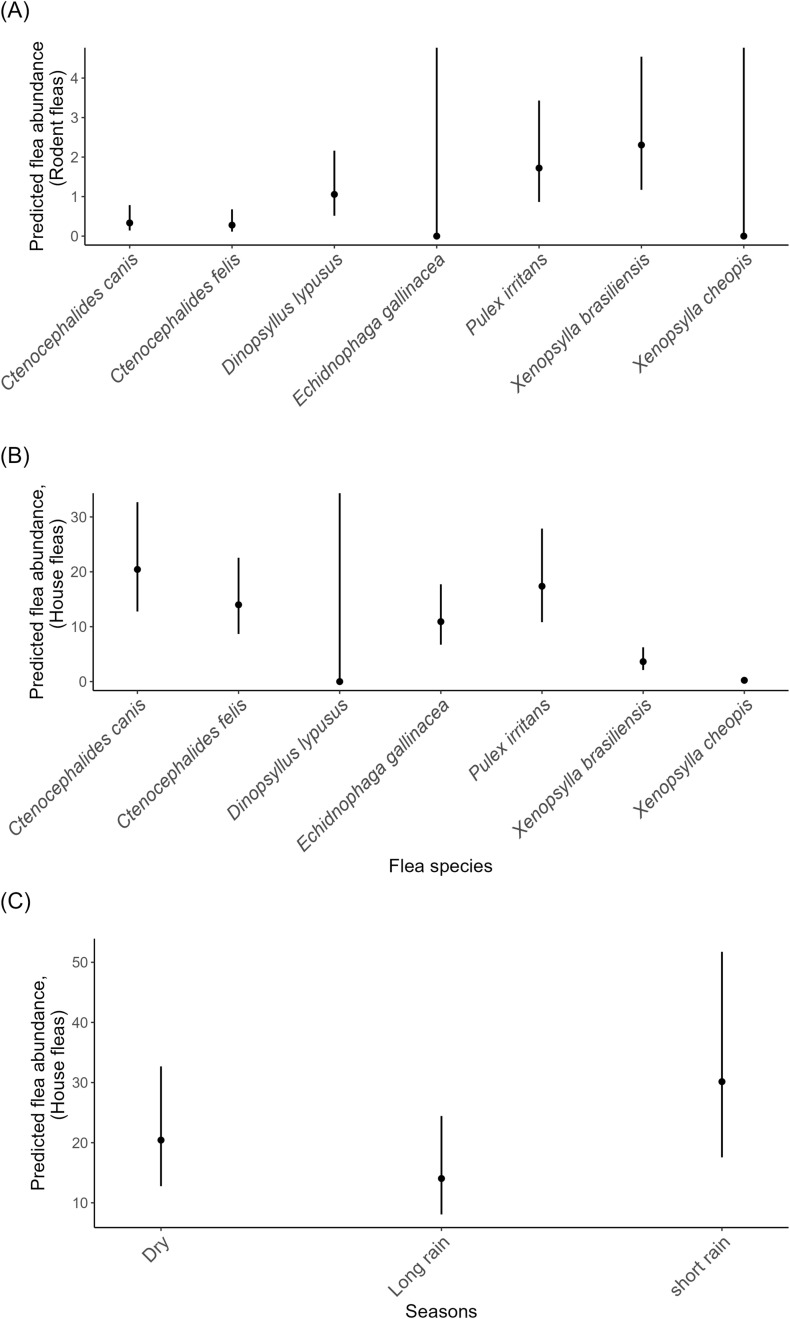
Prediction plots showing (A) the increase of the rodent flea abundance was mostly likely influenced by species identity: *Xenopsylla brasilliensis, Pulex irritans*, and *Dinopsyllus lypusus.* (B) decrease in the Ltrap flea abundance was mostly likely influenced by the species identity: *Xenopsylla brasilliensis, E. gallinacea*, and *X. cheopis* and (C) The abundance of house flea in the house was more likely to be higher during the short rain season. The error bars represent the 95% confidence intervals around the predicted means.

The final best model showed that flea species *X. brasilliensis* (mean = 1.93 ± 0.56SE, p = 0.001), *P. irritans* (mean = 1.64 ± 0.56SE, p = 0.003), and *D. lypusus (*mean = 1.15 ± 0.57SE, p = 0.04) were significantly associated with increased abundance of rodent flea (Fig 5A). On the other hand, *X. cheopis* (mean = -4.48 ± 0.66SE, p < 0.001), *X. brasilliensis* (mean = -1.73 ± 0.34SE, p < 0.001) and *E. gallinacea* (mean = -0.63 ± 0.31SE, p = 0.05) were significantly associated with the decreased abundance of house flea (Ltrap). Moreover, short rain season was mostly associated with the abundance of house flea (mean = 0.39 ± 0.24SE, p = 0.11). In contrast the abundance decreased during the long rain season (mean = -0.37 ± 0.25SE, p = 0.14) compared to the dry season. However, these trends were not statistically significant (Fig 5B and C).

#### Effect of house characteristics on flea abundance.

**Fig 6:**
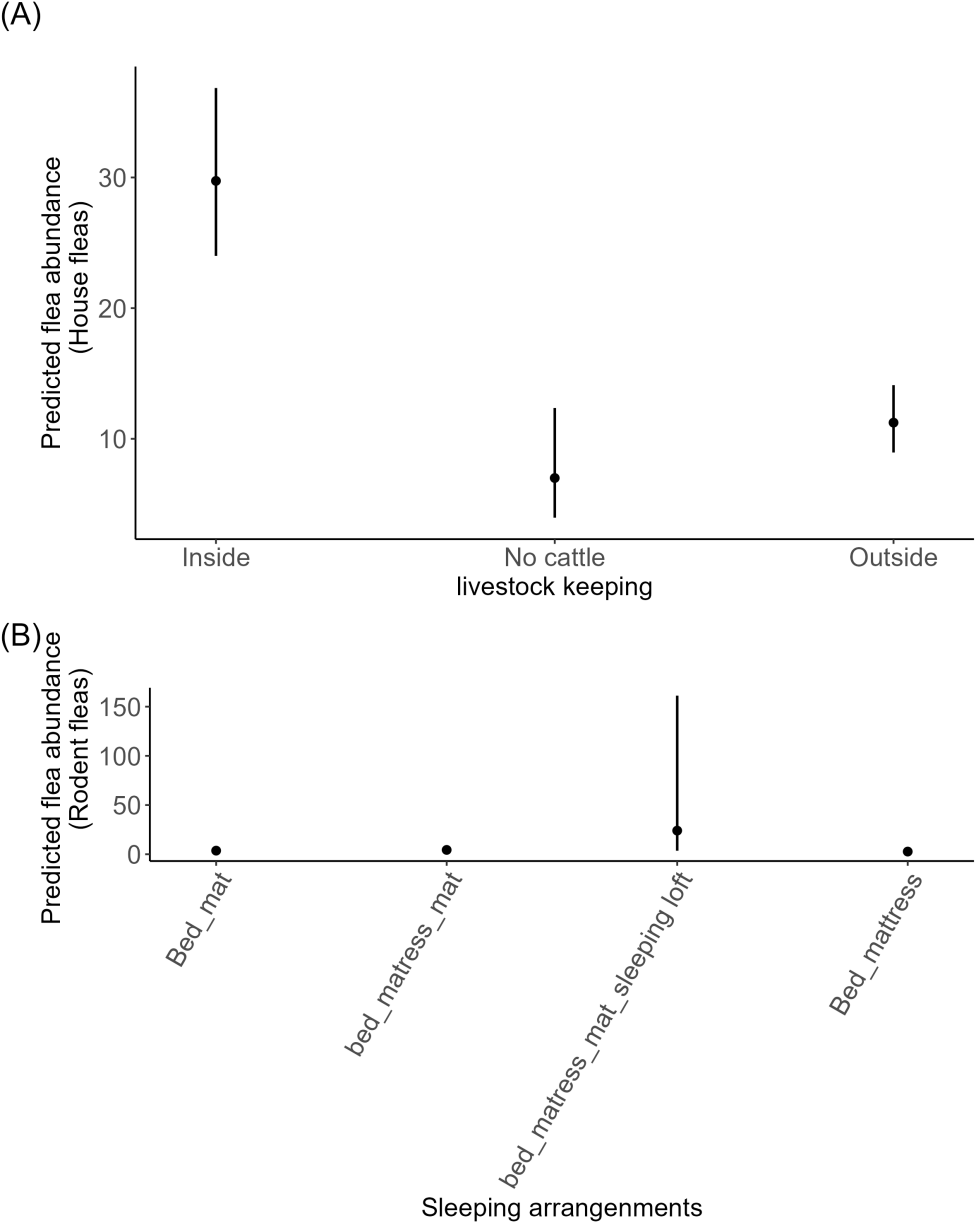
Prediction plots indicate that (A) houses with livestock inside sleeping houses were more likely to have higher abundance of Ltrap fleas compared to houses with no cattle or those with livestock kept outside. (B) Houses with sleeping arrangement sleeping loft show a higher probability of having more rodent fleas compared to houses with sleeping arrangement that has no sleeping loft.

Our final model results from the house flea abundance (Ltrap) indicated that houses with no livestock (mean = -1. 45 ± 0.31SE, p < 0.001) and those maintained livestock outside sleeping houses (mean = -0.97 ± 0.16SE, p < 0.001) was mostly significantly associated with the decrease of house flea abundance compared to houses with livestock kept within sleeping house. Additionally, results from the rodent fleas showed that houses with sleeping arrangement bed, mattress/mat and sleeping loft (mean = 1.88 ± 1.04SE, p = 0.07) had highest probability of increased abundance of rodent flea compared to houses with sleeping arrangement bed mattress alone. However, this increase was not statistically significant (Fig 6).

## Discussion

This study aimed to understand the trends in flea index and flea population from the residential areas located in a district prone to bubonic plague. We found majority of the flea species present in the indoor environment and on the rodent species *R. rattus,* which was mostly dominant abundant amongst other rodent species. Flea species *X. brasilliensis* was the most common species found on the rodent host and the environment. Also, we found *D. lypusus* only on the rodent host and species *C. canis, C.felis* and *X. cheopis* were found only in the substrates. Further, flea indices for rodent and environment fleas varied throughout the sampling period. The rodent flea index showed an increase during March, June and November, while the environment flea index peaked in August and October, remaining low from March to June. Moreover, our GLM results showed species *X.brasilliensis*, *P.irritans* and *D. lypusus* increased the abundance of rodent flea while species *C. canis* increased the abundance of the Ltrap flea. The abundance of Ltrap flea in the house increased during the short rain season. Additionally, we found that houses with no livestock and those maintaining livestock outside the sleeping area, were more likely to experience a decrease in environment flea abundance compared to household keeping livestock inside houses. In contrast, sleeping arrangement such as a sleeping loft were associated with an increased abundance of rodent fleas.

The abundance of the rodent hosts was mostly dominated by the *R.rattus* followed by *M. natalensis*. This may have been attributed to the house characteristics; for instance, houses were very close to farms, crops were stored inside houses and livestock were kept in the same house or in corrals situated near sleeping houses. These provided resource such as food and shelter for these species. It is known that these two species, tend to coexist with human [53,63], harbors *Yersinia pestis* [37,41], and contribute to the persistence of plague in the foci [34,64–66], highlighting that their presence in residential areas may have a potential role in the persistence of the disease in this focus.

Majority of the fleas collected were from the surroundings compared to those found on the rodent hosts. Flea species *X. brasilliensis* was most dominant on rodent hosts and in the Ltrap. Among the rodent host, *R.rattus* had the highest abundance of this flea species, indicating a strong association between this flea species and *R. rattus*. Also, the flea species *D. lypusus* was found only on the rodent species, while *C. canis*, *C.felis* and *X.cheopis* were found only in the Ltrap. Previous studies have shown that *X. brasiliensis* and *D. lyspusus* were the competent vectors for transmitting plague during 2007 outbreak in Mbulu [43]. Additionally, *D. lypusus* is known an important enzootic flea in maintaining *Y.pestis* [65,67] and a bridging vector transmitting the disease from the sylvatic environments to domestic environments [68]. The observed relationship between these flea species and rodent hosts, their role as competent vector for *Y.pestis* not only highlight their potential to sustain and transmit plague in this rural setting but also shows the complex dynamics of flea-borne disease transmission that may be existing in this area. The presence of fleas in the Ltrap not associated with the rodent hosts suggests two possibilities; one, some may have been seeking new hosts due to fluctuations in rodent dynamics, two, others were probably distinct population minimizing inter-specific competition example the *C. felis*. The ability of this species to thrive off-host can be related to the domestic animals’ chambers and the accumulation of organic waste in the houses. While they feed on blood, they can also develop in environments enriched with nutrients from host waste allowing them to persist in the residential areas [20].

The total flea indices for both rodent fleas and Ltrap fleas exceeded 1, indicating higher chances of plague transmission risks. This corroborates with other studies [69,70], indicating how higher flea indices correlate with greater likelihood for epizootic spread and human exposure to the plague pathogen *Y.pestis*. During the sampling period, we observed an increase in rodent flea index in March (long rain season), June (dry season), and November (short rain season), all of which were the beginning of the seasons. This increase may have been influenced by the environmental conditions such as the close proximity to houses and farmlands [47]. Our previous study indicated rodent abundance was high during dry season and species richness peaked both during the dry and short rain seasons in the foci [53,71]. This condition is further supported by the significant positive correlation observed between rodent and the house flea abundance. Thus, as rodent abundance increases, house flea abundance also tends to increase, suggesting that this relationship could be enhancing the flea indices and contributing to the dynamics of plague transmission risks.

House fleas index increased from June (dry season) reaching its peak in October and started declining in November (short rain season). This increase may have been contributed by human activities during these months. Our field observation indicated that, animals are often taken out to graze only during the rainy season, while they remain indoor during dry season and fed hay and stalks harvested from the farms [47], which may have created a microhabitat conducive to flea development. The accumulation of organic material provides food and shelter for flea larvae, enhancing their survival rates [72]. Organic matter such as hay, animal waste and plant debris create favorable habitats that supports flea development and reproduction. Additionally, grazing practices may facilitate contacts between livestock and wild rodents, promoting the transfer of fleas between species and potentially increasing the overall flea population in the environment [73].

Furthermore, we observed that high flea index in the dry season and short rain seasons were dominated by the species *C. canis*, *P.irritans* and *C. felis* all of which have high probability of increasing the house flea abundance. This finding corroborates other studies conducted elsewhere in Mongolia, which revealed that flea index to increase during drier conditions and more sunshine [74]. The indoor environment during the dry season likely provided survival micro-climatic conditions conducive for flea immature stages [75,76]. Specifically, the accumulation of organic material from hay and animal waste can create a warm and sheltered habitat that supports flea larvae development. Moreover, the indoor humidity levels may reach a threshold optimal for flea survival, particularly when outdoor conditions are less favourable. During the long rainy season, excessive humidity may be detrimental to flea larvae, leading to higher mortality rates [76,77]. Our previous study [47] observed that most people experience flea bites during the dry season, consistent with the observed higher house flea index during this season. On the other hand, studies from this area revealed that plague transmission is seasonal, occurring between November and March and peaking between December and February [33,37], which is a short rainy season to a long rainy season. This suggest that while the peak of flea biting activity occurs during dry season (June to Sept), the movement of livestock to grazing areas during the rainy seasons may likely increase chances of livestock bringing bacteria from wild rodents into close contact with humans, potentially enhancing plague transmission risks despite experiencing lower biting frequency during this period.

Moreover, we observed increase in Ltrap fleas in houses keeping livestock inside household. This finding aligns with other studies [44,78], which indicate that livestock within houses offer adequate food and shelter for flea flourishing. Additionally, grazing activities in the study area may contribute the spread of fleas in residential settings, as dogs and cattle can act as bridging hosts, carrying fleas from the wild to the domestic areas. This increases the risk of spreading flea pathogens to the human settlements. For instance, the flea species *C. canis*, has been shown to serve as a bridge between wildlife and human settlements [79] and is known as a competent vector for zoonotic pathogen including *Rickettsia felis* [80]. These findings highlight the need for effective public health strategies and a broader understanding of the overlap of these pathogens in the study area. The study area has been shown to harbor *Y. pestis* in rodents [41,43], has a high abundance of rodent reservoir and their flea vector, [40,53,71,62] and has also demonstrated the presence of *Rickettsia typhi* in rodent fleas [81].

Houses with sleeping arrangements that included a bed, mattress, mat and a loft were associated with increased rodent flea abundance. In the study area, many houses were constructed from mud and thatch, having loft where crops were stored and mats were used for sleeping (Fig 2). The loft area was characterized by wooden floor, was dark and less disturbed compared to other parts of the house. The lack of disturbance likely created a suitable microhabitat for flea survival and development, contributing to the observed increase in flea abundance. However, trapping was conducted only on the lower levels of the house which may limit our understanding of flea populations residing in the loft. Poor house quality likely created ideal conditions for rodent infestations, hence their fleas. This finding corroborates other research that has shown building designs and materials, including lofts provide hiding and nesting places for rodents, enhancing their persistence in homes [45]. [80]. Usually, fleas thrive in an environment with abundant rodent hosts as they provide necessary conditions for flea survival [82]. Therefore, poor sanitation and house arrangements may have contributed to the increased population of rodent flea in our study area, a trend that was also observed in other rural areas in Madagascar [28,46].

This study reveals critical insights into flea index dynamics within rural settings in Tanzania. It demonstrates the interplay between rodent populations, flea vectors, and human living conditions on enhancing the likelihood for plague persistence and transmissions. These findings have public health implications, Firstly, the seasonal patterns of flea abundance suggest optimal timing for vector control interventions, particularly during transitional periods between seasons when flea indices peak. Secondly, increased rodent flea abundance in the houses that had sleeping arrangements bed, mattress, mats and loft as well as increased Ltrap fleas in houses keeping livestock inside household indicate that improvements to the household practices and sleeping arrangements to reduce the flea indices and disease risks is an increasing priority in rural Tanzania. While our finding has important public health implications, we acknowledge that real-time monitoring of indoor temperature and humidity could offer more insights and enhance our understanding of flea population dynamics. Future research should focus on examining *Y.pestis* presence and dynamics across climatic and environmental gradients to establish a more direct link between disease persistence and transmission risks.
